# Climate warming causes life-history evolution in a model for Atlantic cod (*Gadus morhua*)

**DOI:** 10.1093/conphys/cou050

**Published:** 2014-11-04

**Authors:** Rebecca E. Holt, Christian Jørgensen

**Affiliations:** 1 Department of Biology, University of Bergen, PO Box 7803, 5020 Bergen, Norway; 2 Uni Research, PO Box 7810, 5020 Bergen, Norway

**Keywords:** Adaptation, behaviour, bioenergetics, climate, respiratory physiology

## Abstract

Climate change influences the marine environment, with ocean warming being the foremost driving factor governing changes in the physiology and ecology of fish. At the individual level, increasing temperature influences bioenergetics and numerous physiological and life-history processes, which have consequences for the population level and beyond. We provide a state-dependent energy allocation model that predicts temperature-induced adaptations for life histories and behaviour for the North-East Arctic stock (NEA) of Atlantic cod (*Gadus morhua*) in response to climate warming. The key constraint is temperature-dependent respiratory physiology, and the model includes a number of trade-offs that reflect key physiological and ecological processes. Dynamic programming is used to find an evolutionarily optimal strategy of foraging and energy allocation that maximizes expected lifetime reproductive output given constraints from physiology and ecology. The optimal strategy is then simulated in a population, where survival, foraging behaviour, growth, maturation and reproduction emerge. Using current forcing, the model reproduces patterns of growth, size-at-age, maturation, gonad production and natural mortality for NEA cod. The predicted climate responses are positive for this stock; under a 2°C warming, the model predicted increased growth rates and a larger asymptotic size. Maturation age was unaffected, but gonad weight was predicted to more than double. Predictions for a wider range of temperatures, from 2 to 7°C, show that temperature responses were gradual; fish were predicted to grow faster and increase reproductive investment at higher temperatures. An emergent pattern of higher risk acceptance and increased foraging behaviour was also predicted. Our results provide important insight into the effects of climate warming on NEA cod by revealing the underlying mechanisms and drivers of change. We show how temperature-induced adaptations of behaviour and several life-history traits are not only mediated by physiology but also by trade-offs with survival, which has consequences for conservation physiology.

## Abstract (precis)

We provide a state-dependent energy allocation model to assess the impacts of climate warming on the life-history evolution and behaviour of Atlantic cod, specifically the North-East Arctic stock (NEA). Model predictions show the response is positive for this stock, individuals grow quicker and are more fecund, exhibiting increased foraging behaviour. We reveal the underlying mechanisms and drivers of change in NEA cod, which has important implications for conservation physiology.

## Introduction

Virtually all fish are ectotherms or thermal conformers, whereby their internal temperature is determined by the surrounding water (Jobling, 1996; Clark *et al.*, 2003). This is potentially problematic, as most of the heat from climate warming is stored in the ocean, and temperatures are projected to increase even under the most restrictive emission scenarios (IPCC, 2013). Even small changes in temperature can directly influence biochemical rates and enzyme kinetics (Hochachka and Somero, 2001; Pörtner, 2001, 2010). Temperature is therefore considered to be a key driver of changes in the physiology and ecology of fish (Clark *et al.*, 2003; Pörtner and Peck, 2010). Studies have documented effects of climate warming in terms of species' range shifts (Perry *et al.*, 2005), population collapses and local extinctions (Pörtner and Knust, 2007), as well as changes in phenology (Wiltshire and Manly, 2004). These studies tend to focus on populations, communities and ecosystems. However, biological responses to climate change will ultimately be driven by mechanisms at the individual level, such as physiology and behaviour and their effects on growth, survival and reproduction (Rijnsdorp *et al.*, 2009). In turn, these individual-level responses have consequences for populations and beyond. The species should, furthermore, be viewed as dynamic in time (non-static), as traits may potentially be affected by phenotypic plasticity in the short term and by evolution in the longer term.

A primary mechanism through which temperature change is expected to affect marine fish is respiratory physiology, because it provides limits for bioenergetics and has consequences for performance (Priede, 1985), as described by the theory of oxygen and capacity limitation of thermal tolerance (OCLTT; Pörtner and Knust, 2007; Pörtner *et al.*, 2008; Pörtner, 2010). The OCLTT theory takes its starting point at the Fry (1971) paradigm, which describes how standard metabolic rate (SMR) increases exponentially with temperature, whereas the maximal capacity for oxygen uptake (maximal aerobic metabolism; AMR) reaches an asymptote due to morphological and physiological constraints (Pörtner and Peck, 2010). The difference between AMR and SMR is the aerobic scope or scope for activity. The aerobic scope typically increases with temperature up to an optimum, after which it declines progressively with further increases in SMR. At the critical temperature, the individual uses all of its aerobic scope to meet the oxygen demands of SMR, leaving no aerobic scope for other processes. Aerobic scope is critical for fitness because it defines the scope for metabolic processes related to growth, locomotion and reproduction (Pörtner and Peck, 2010); therefore, moderate decreases in aerobic scope can translate into changes in these key life processes and may cause a loss in growth, reproduction or survival (Pörtner and Peck, 2010). Other potential effects of climate change include ocean acidification, hypoxia or ocean stratification (Roessig *et al.*, 2004). The OCLTT theory relates to these by describing how hypoxia, acidosis and other environmental drivers may impact the AMR–temperature curve, thus integrating multiple stressors through their effects on one mechanism (Pörtner, 2010).

Temperature is, however, the foremost driving factor governing changes in the physiology and ecology of fish (Roessig *et al.*, 2004; Carey and Zimmerman, 2014). In this paper, we therefore focus on temperature and provide a mechanistic model based on bioenergetics and respiratory physiology to derive predictions of optimal temperature-induced responses for life histories and behaviour of Atlantic cod (*Gadus morhua*), specifically the North-East Arctic stock, hereafter referred to as NEA cod, which inhabits the Barents Sea in the Northeast Atlantic and spawns along the Norwegian coast. The NEA cod are typically depicted as having ‘slow’ life histories; they grow slowly at ∼10 cm year^−1^ and adults are often larger than 1 m, they typically mature at 6–10 years of age and may live up to 25 years, and as such, they are sensitive to high mortality rates (Wiedmann *et al.*, 2014). The NEA cod is a commercially important stock, and large amounts of experimental and survey data enable validation of model predictions. Atlantic cod is a species for which temperature responses have been studied extensively (Brett, 1956; Jobling, 1988). Studies describe temperature-dependent increases in length (Michalsen *et al.*, 1998; Björnsson *et al.*, 2007) and stock-specific differences in weight (Brander, 1995) as well as optimal temperatures for growth (Björnsson *et al.*, 2001; Pörtner, 2001; Pörtner *et al.*, 2008; Pörtner and Peck, 2010). Increased temperatures are suggested to have both positive and negative impacts on cod spawning stock biomass (Ottersen *et al.*, 2006) and on recruitment (Planque and Frédou, 1999; Drinkwater, 2005). Cod is also one of the few species for which temperature dependence is known for both resting and maximal metabolic rates (Claireaux *et al.*, 2000), as well as optimal (Pörtner, 2001; Pörtner *et al.*, 2008) and critical temperatures of respiration, making it an ideal species for which to model the effects of temperature on metabolism and its consequences for organismal performance, life histories and fitness.

Our aim is to provide predictions for the effects of climate warming on adult life-history strategies, behaviour and population dynamics of NEA cod in the Barents Sea, using a state-dependent mechanistic model. Dynamic state-dependent modelling approaches have been used to study the effect of temperature on life-history strategies in salmonids (Mangel, 1994) and steelhead trout (*Oncorhynchus mykiss*; Satterthwaite *et al.*, 2009). Dynamic state-variable approaches allow rich organism detail and inclusion of conditional strategies while using evolutionary methodologies to find optimal responses to a given environment. Our model follows the same framework and concepts, but extends beyond earlier state-dependent models for cod (Jørgensen and Fiksen, 2006, 2010; Jørgensen and Holt, 2013) by being more explicit about physiological detail, in particular aerobic respiration and the oxygen budget.

With our model, we show how optimal behavioural and life-history strategies vary with temperature for NEA cod, and how physiology, in particular respiratory constraints and oxygen budgeting, together with trade-offs related to survival act as both constraints and drivers of change. Our findings suggest that one should view the species physiology and ecology as a whole when predicting the effects of climate warming, being aware that organisms possess a suite of traits that have co-evolved.

## Materials and methods

### Model description

**Figure 1:**
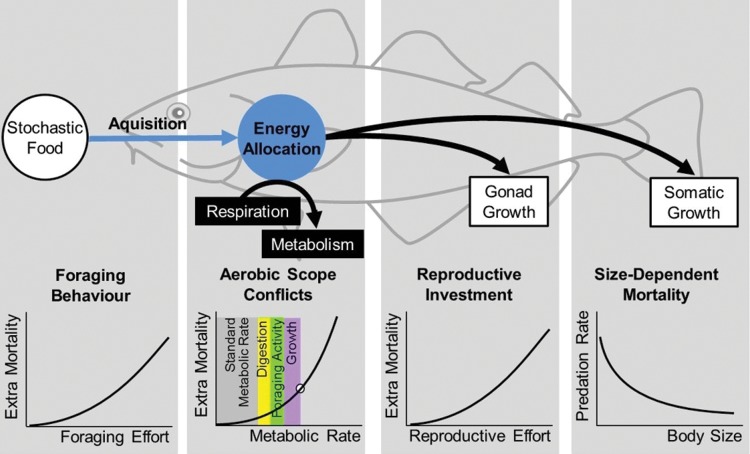
Schematic overview of the state-dependent mechanistic model describing energy allocation in response to climate warming for North-East Arctic cod. Arrows indicate energy flow, with respiratory constraints and oxygen budget highlighted by the black boxes. Central trade-offs with survival are shown as graphs for foraging behaviour, aerobic scope, reproductive investment and size-dependent predation. Blue indicates strategies found by evolutionary optimization: foraging behaviour and energy allocation.

We use a state-dependent energy allocation model in which optimal fish life-history strategies are found by optimization using dynamic programming (Houston and McNamara, 1999; Clark and Mangel, 2000). The current model (Fig. 1) is an extension of the models developed by Jørgensen and Fiksen (2010) and Jørgensen and Holt (2013), with greater emphasis on respiratory physiology and temperature dependence and with stochastic food availability. The model includes a number of trade-offs that reflect key physiological and ecological processes; several of the trade-offs involve survival, and as a consequence, natural mortality has been split into different components (Fig. 1, Table 1). An optimal life-history strategy comprises the values for the foraging behaviour (*φ*) and the energy allocation strategy (*α*) that maximize the expected future reproductive output, and optimal strategy values are found for each combination of the individual states age (*A*), length (*L*) and the current food environment (*E*). The optimal strategy is then simulated in a population to produce the results shown. Maturation, reproduction, foraging behaviour and growth are thus emerging from evolutionary optimization given constraints from physiology and ecology. To find adaptive responses to climate warming, we step-wise re-run the model to find new optimal life-history strategies in a new and warmer environment.

**Table 1:** Parameters used in the model for the life-history evolution and behaviour of the North-East Arctic stock of cod in response to climate warming

Symbol	Description	Units
*φ*	Strategy variable: foraging behaviour	—
*α*	Strategy variable: energy allocation	—
*A*	Age	years
*L*	Length	cm
*E*	Stochasticity of the food environment	—
σ_E_	Standard deviation (stochasticity of the food environment)	—
*W*	Somatic weight	kg
*G*	Gonad weight	kg
*N*	Net resources available for growth and reproduction	J year^−1^
*H*	Foraging intake function	J year^−1^
*T*	Temperature	°C
$\tilde{T}(y)$	Temperature seasonality as function of time within year	°C
*B*_SMR_	Standard metabolic rate	J year^−1^
*B*_φ_	Energetic cost of foraging behaviour	J year^−1^
*B*_SDA_	Energetic cost of digestion	J year^−1^
*B*_M_	Energetic cost of spawning migration	J year^−1^
*U*_opt_	Optimal swimming speed	BL s^−1^
*C*_M_	Cost of transport	J km^−1^
*B*_M_	Energetic cost of migration	J year^−1^
*V*_max_	Maximal oxygen uptake	J year^−1^
*B*_growth_	Energetic cost of growth	J year^−1^
*V*	Actual oxygen consumption	J year^−1^
*M*_predation_	Size-dependent predation mortality	year^−1^
*M*_foraging_	Foraging mortality	year^−1^
*M*_reproduction_	Mortality related to reproduction and gonads	year^−1^
*M*_respiration_	Mortality related to oxygen budgeting conflict and aerobic scope	year^−1^
*Q*	Gonadosomatic index	—
*S*	Annual survival probability	year^−1^
*Z*	Total mortality	year^−1^
*R*	Expected future reproductive output	kg

Dimensionless variables are assigned ‘—’ in the column for units.

#### Gonadal and somatic growth

The model is an energy allocation model whereby the net available resources (*N*; in joules per year) are channelled towards growth of somatic body mass (*W*; in kilograms) or gonads (*G*; in kilograms) according to the energy allocation strategy (*α*) taking values between zero and one, as follows:

(1) $$\frac{dW}{dt}=\frac{(1-\alpha )N}{x_{W}}$$

(2) $$\frac{dG}{dt}=\frac{\alpha N}{x_{G}}$$

where *x*_W_ and *x*_G_ (in joules per kilogram) are the energy density of somatic and gonadal cod tissue (wet weight; Holdway and Beamish, 1984). Net energy intake (*N*) summarizes the energy flow and accounts for all costs and biochemical conversions. The parameter *G* is interpreted broadly as reproductive investment and includes gonadal tissue plus all energetic costs associated with reproduction, such as behaviour and migrations. It is assumed that body length (*L*; in centimetres) relates to somatic weight as *W* = *kL*^3^. As a fitness measure, we optimize expected lifetime production of gonadal mass.

The previous versions of this model (Jørgensen and Fiksen, 2010; Jørgensen and Holt, 2013) did not model bioenergetics or the oxygen budget explicitly, but assumed that *N* in equations (1) and (2) represented a net energy flow as a function of foraging behaviour. For the present study, our focus is climate effects, and to incorporate temperature dependence in a physiologically consistent way there was a need (i) to make the energy budget explicit because different energetic costs might be affected by temperature in different ways, and (ii) to include maximal aerobic respiration rate and an explicit oxygen budget to include the perspectives of the OCLTT theory (Pörtner, 2010), because respiratory constraints are thought to be one of the main mechanisms affecting climate change responses in marine organisms.

#### Bioenergetics overview

The model is based upon the Wisconsin bioenergetics framework (Hewett and Johnson, 1992), but is more rigorous towards the underlying physiology as detailed below. The overall energetic flow is summarized by the equation for net energy intake *N* (in joules per year; each element is described further below):

(3) $$\begin{array}{ll} & N=[H(\varphi ,E,W)-B_{\mathrm{SMR}}(T,W+G)\\ & \;-B_{\varphi }(\varphi ,W)-B_{\mathrm{SDA}}(H)]c_{R} \end{array}$$

where *H* (in joules per year) is the foraging intake function, which determines energy ingestion, depending on the foraging behaviour (*φ*), temperature (*T*), the current value (*E*) of the stochastic food environment, and scales allometrically with somatic body mass (*W*). The remaining terms are energetic costs (in joules per year), as follows: *B*_SMR_ is standard metabolic rate (SMR), which depends on temperature and total body mass (*W* + *G*); *B*_φ_ is the energetic cost of the foraging behaviour (*φ*), being a function of somatic body mass; and *B*_SDA_ is the energetic cost of digestion (specific dynamic action, or SDA), which is a proportion of foraging intake. In addition there is a cost (*B*_M_) of the spawning migration if the individual reproduces, subtracted directly from gonads at the time of spawning, where swimming costs scale with total body mass. Each of these is described below. The net energy flow is then converted to new tissue with efficiency *c*_R_.

The individual's food intake is formulated as follows:

(4) $$H(\varphi ,E,W)=(E\cdot \varphi )\cdot B_{\mathrm{SMR}}(T_{0},W)$$

Here the first part in parentheses determines the energetic foraging rate. The term *E* (dimensionless) introduces the stochasticity of the food environment and is a random value from a normal distribution with a mean of one and a standard deviation of σ_E_. The effect of *E* is to make the current food intake higher or lower than the long-term average. Finally, we scale intake to SMR to make interpretation of the behavioural trait *φ* more intuitive, e.g. *φ* = 2 implies that the fish's foraging intake corresponds roughly to 2 · *B*_SMR_ of a lean fish at the standard temperature (*T*_0_). Note that foraging scales with somatic body mass, not total body mass including gonads, as this better represents the strong effect of length in determining predator–prey interactions in aquatic systems.

#### Energy budget

Standard metabolic rate (*B*_SMR_) is modelled using allometric scaling with body mass and the Arrhenius function of temperature dependence, adapted from Clarke and Johnston (1999), as follows:

(5) $$B_{\mathrm{SMR}}(T,W+G)=c_{\mathrm{SMR}}f(T)(W+G{)}^{b}$$

where *c*_SMR_ (in joules per kilogram per year) is a species-specific metabolic coefficient and *b* the scaling exponent for metabolic rate; note that gonads (*G*) contribute to standard metabolic rate in the same way as somatic body mass (*W*). The function *f*(*T*) is an Arrhenius relationship for temperature dependence given by:

(6) $$f(T)=\exp\left\{c_{T1}-\left[\frac{c_{T2}}{\left(T+273.15\right)}\right]\right\}$$

where *c*_T1_ and *c*_T2_ (in kelvin) are constants and *T* (in degrees Celsius) the experienced temperature. The scaling exponent was held constant with temperature, although recent literature suggests that scaling exponents for SMR may decrease at warmer temperatures (Killen *et al.*, 2010; Ohlberger *et al.*, 2012); however, this has not yet been documented for cod.

We assume that foraging activity incurs energetic costs of swimming that are independent of temperature, as follows:

(7) $$B_{\varphi }(W)=c_{\varphi }\varphi \;B_{\mathrm{SMR}}(T_{0},W)$$

Here *c*_φ_ (in joules per year) is the coefficient for the energetic cost of foraging behaviour and *T*_0_ is the temperature at which the cost is specified (the current mean temperature in the Barents Sea).

The model assumes that a fixed fraction (*c*_SDA_) of ingested energy (*H*) is lost as specific dynamic action (*B*_SDA_), which includes the mechanical costs of ingestion and digestion as well as biochemical costs associated with absorption and assimilation, as follows:

(8) $$B_{\mathrm{SDA}}(H)=c_{\mathrm{SDA}}\cdot H$$

The costs above are subtracted from intake and corrected for the efficiency of energy conversion to obtain the net energy available for growth, *N* (equation 3). Some authors include costs of growth within SDA; in our model, there are separate costs of biochemical processes when intake is used for growth (through efficiency loss, *c*_R_) as opposed to when ingested energy is just covering SMR, digestion and locomotion.

Finally, Atlantic cod makes annual migrations to and from its spawning grounds, and these annual migrations entail direct costs in terms of both energy and time. We model migration based on Ware (1978), where optimal swimming speed [*U*_opt_; in body lengths (BL) per second] is a function of length as $U_{\mathrm{opt}}=c_{U}\cdot L^{b_{2}}$, from which the cost of transport (*c*_M_; in joules per kilometre) is calculated as $c_{M}=c_{\mathrm{COT}}L^{b_{3}}U_{\mathrm{opt}}^{b_{4}}$. The migration cost [*B*_M_(*W*); in joules per year] is then found by multiplying with twice (round trip) the distance of the spawning migration (*D*_M_; in kilometres), as follows:

(9) $$B_{M}(W)=c_{M}\cdot 2\cdot D_{M}$$

The cost of migration is subtracted from gonads at the time of spawning. The model allows allocation of energy to reproduction by building gonadal mass throughout the year, but spawning is a distinct event. If no allocation to reproduction occurs, no migration takes place, and the fish remains at the feeding grounds. To avoid complicated calculations of the duration of migration, we keep migration costs outside the aerobic budget explained below.

#### Oxygen budget

In addition to considering an individual's energy budget, our model also considers its aerobic oxygen budget. Illustrative experimental evidence for such a link was obtained for Atlantic silversides (*Menidia menidia*), where individuals with higher voluntary meal size and faster growth rates were more readily predated upon because of their reduced swimming performance (Billerbeck *et al.*, 2001; Lankford *et al.*, 2001), in turn due to elevated oxygen requirements resulting from higher digestion rates (Lankford *et al.*, 2001; Arnott *et al.*, 2006). To include such effects, the model sums all metabolic processes, and the degree to which the aerobic scope is used has consequences for survival.

Maximal oxygen uptake (*V*_max_; in joules per year) is modelled using allometric scaling with body mass and is temperature dependent, adapted from Claireaux *et al.* (2000). The resulting relationship for *V*_max_(*T*,*W*) is as follows:

(10) $$V_{\max}(T,W)=(V_{1}T^{\left(-V_{2}\;T+V_{3}\right)}+V_{4})W^{b}$$

with *v*_1_–*v*_4_ being parameters given in Table 2; the relationship is shown graphically in Supplementary Fig. S1. The *V*_max_ scaling exponent is the same as used for *B*_SMR_ (equation 5). Note that only somatic mass is used here; gonadal mass does not affect the scaling of oxygen uptake but does contribute to oxygen consumption through SMR.

**Table 2:** Parameters used for the North-East Arctic stock of cod in a model for state-dependent energy allocation in response to climate warming

Symbol	Description	Value	Units	Source
*x*_W_	Energy density of somatic tissue (wet weight)	4.62 × 10^6^	J kg^−1^	Holdway and Beamish (1984)
*x*_G_	Energy density of gonadal tissue (wet weight)	6.93 × 10^6^	J kg^−1^	Holdway and Beamish (1984)
*k*	Condition factor of somatic weight	0.95	kg^−1^	
*c*_R_	Conversion efficiency	0.5	—	
*c*_SMR_	Standard metabolic rate coefficient	4.18 × 10^6^	J kg^−1^ year^−1^	Clarke and Johnston (1999)
*b*	Scaling exponent for metabolic rate	0.70	—	
*c*_T1_	Standard metabolic rate temperature function	15.7	K	Clarke and Johnston (1999)
*c*_T2_	Standard metabolic rate temperature function	5020	K	Clarke and Johnston (1999)
*c*_φ_	Energetic cost of foraging behaviour coefficient	0.15	J year^−1^	
*T*_0_	Current mean habitat temperature in Barents Sea	4	°C	Drinkwater (2005)
*c*_SDA_	Energetic cost of digestion	0.17	J kg^−1^ year^−1^	Hansson *et al.* (1996)
*c*_U_	Optimal cruising speed parameter for pelagic fish	0.138	s^−1^	Ware (1978)
*b*_2_	Scaling factor for optimal cruising speed for a pelagic fish	0.43	—	Ware (1978)
*c*_COT_	Cost of transport coefficient	4.18 × 10^1^	J km^−1^	Ware (1978)
*b*_3_	Length scaling factor	1.02	—	Ware (1978)
*b*_4_	Swimming speed scaling factor	2.42	—	Ware (1978)
*D*_M_	Spawning migration distance	780	km	Jørgensen and Fiksen (2006)
*v*_1_	Maximal oxygen uptake parameter	4.58 × 10^5^	J year^−1^	Claireaux *et al.* (2000)
*v*_2_	Maximal oxygen uptake parameter	0.015	°C	Claireaux *et al.* (2000)
*v*_3_	Maximal oxygen uptake parameter	1.062	°C	Claireaux *et al.* (2000)
*v*_4_	Maximal oxygen uptake parameter	7.96 × 10^5^	J year^−1^	Claireaux *et al.* (2000)
*c*_predation_	Predation mortality coefficient	0.33	year^−1^	
*d*	Predation mortality exponent	0.75	—	McGurk (1986)
*c*_foraging_	Foraging mortality coefficient	0.03	year^−1^	
*f*_1_	Foraging mortality exponent	3	—	
*q*_ref_	Gonadosomatic index at which *M*_reproduction_ = *M*_predation_	0.10	—	
*P*	Gonad mortality exponent	2.5	—	
*c*_respiration_	Respiration mortality coefficient	11	year^−1^	
*u*	Respiration mortality exponent	3	—	
*M*_fixed_	Size-independent mortality	0.07	year^−1^	
*T*_a_	Temperature amplitude	1.04	°C	ICES (2012)
*T*_p_	Temperature peak	0.66	year	ICES (2012)
*F*	Fishing mortality	0.17	year^−1^	

Oxygen consumption is the sum of all aerobic metabolic processes, as follows:

(11) $$\begin{array}{ll} & V(T,\varphi ,H,W,G)=B_{\mathrm{SMR}}(T,W+G)\\ & \;\;\;\;+B_{\mathrm{SDA}}(H)+B_{\varphi }(\varphi ,W)+B_{\mathrm{growth}} \end{array}$$

Here *B*_growth_ is the metabolic work associated with energy conversion (*c*_R_) related to somatic and gonadal growth and needs to be accounted for in the oxygen budget as it requires oxygen, as follows:

(12) $$\begin{array}{ll} & B_{\mathrm{growth}}=(1-c_{R})[H(\varphi ,T,E,W)-B_{\mathrm{SMR}}(T,W+G)\\ & \;\;-B_{\varphi }(W)-B_{\mathrm{SDA}}(H)] \end{array}$$

To use definitions in the fish physiology literature, aerobic scope is given as *V*_max_ – *B*_SMR_ (Supplementary Fig. S1), and this can be allocated to key life processes, such as growth and reproduction. The free scope is *V*_max_– *V*, and the factorial scope would be *V*_max_/*B*_SMR_.

#### Trade-offs with survival

Many of the physiological and phenotypic traits included in the bioenergetics description above have consequences for survival; total mortality (*Z*; per year) in this model is therefore split into several components, as follows:

(13) $$\begin{array}{ll} & Z=M_{\mathrm{predation}}(L)+M_{\mathrm{foraging}}(\varphi ,L)+M_{\mathrm{reproduction}}(G,L)\\ & \;+M_{\mathrm{respiration}}(V,V_{\max},L)+M_{\mathrm{fixed}}+F \end{array}$$

The different components (all with units of per year) relate to size-dependent predation (*M*_predation_), foraging mortality (*M*_foraging_), gonads and reproduction (*M*_reproduction_), oxygen budgeting and aerobic scope (*M*_respiration_), a fixed mortality due to disease and disaster (*M*_fixed_) and fishing (*F*) and are further described in detail by Jørgensen and Fiksen (2010) and Jørgensen and Holt (2013), as well as below. Annual survival probability (*S*) in year *t* is calculated as follows:

(14) $$S=\exp\left(-\int_{j=t}^{t+1}Z(j)\right)$$

Predation mortality is predominantly size dependent and declines with increasing body size (McGurk, 1986; Gislason *et al.*, 2010), as follows:

(15) $$M_{\mathrm{predation}}(L)=c_{\mathrm{predation}}L^{-d}$$

A central assumption is that the scaling of predation mortality influences the size-dependent scaling of several of the other components of natural mortality, as these involve exposure to predators and eventually the risk of being consumed (Jørgensen and Holt, 2013).

Mortality related to foraging is assumed to be a power function of foraging behaviour (*φ*), as follows:

(16) $$M_{\mathrm{foraging}}(\varphi )=c_{\mathrm{foraging}}\cdot \varphi^{f_{1}}\cdot M_{\mathrm{predation}}(L)$$

The interpretation is thus that a behavioural increase in foraging rate is possible but only by accepting an ever-accelerating mortality rate.

Mortality related to reproduction is caused by the increased energetic burden and decreased locomotory ability when carrying large gonads (Ghalambor *et al.*, 2004). In addition, this mortality component includes risks from exposure to predation during mate search, courtship and spawning. We assume that mortality related to reproduction (*M*_reproduction_) increases with reproductive investment as a function of gonadosomatic index [*Q* = *G*/(*W* + *G*); dimensionless] and that it follows the same size dependence as predation, as follows:

(17) $$M_{\mathrm{reproduction}}(G,L)={\left(\frac{Q}{q_{\mathrm{ref}}}\right)}^{p}\cdot M_{\mathrm{predation}}(L)$$

The sum of all aerobic metabolic activities needs to fit within the organism's *V*_max_. Using the following relationship, we then calculate the ensuing expected mortality from oxygen budgeting conflicts:

(18) $$M_{\mathrm{respiration}}=c_{\mathrm{respiration}}{\left(\frac{V}{V_{\max}}\right)}^{u}\cdot M_{\mathrm{predation}}(L)$$

The last component of natural mortality is a baseline or unavoidable mortality (*M*_fixed_), a constant component that affects all individuals independent of their state or actions, and which can be interpreted to include, for example, disease or environmental catastrophes. For the sake of presenting optimized life histories that resemble the current situation, we also add a baseline fishing mortality (*F*). We include *F* as a fixed mortality rate that does not vary with individual size or state, although we recognize that fishing mortality may vary particularly with size in the natural environment. The value of *F* was chosen depending on the intensity and duration of the stock's exploitation history and to fit predicted growth and maturation trajectories to recent observations. In the model, only maximal oxygen uptake (*V*_max_) and standard metabolic rate (*B*_SMR_) vary with temperature; all temperature relationships in our results are consequences ensuing from these two temperature-dependent processes. In particular, the stochastic food environment and fishing mortality do not vary with temperature.

### Optimization and simulations

We used optimization to find optimal strategies for energy allocation and behaviour under climate warming scenarios. The model was initialized at the current mean temperature in the Barents Sea and then simulated under increasing temperatures. In the model, we focus on adult life stages; fish enter the model at age 1 year, and we make no assumptions that vary larval growth or survival. Numerically, strategies are found for discrete values of the individual states. Although the strategies are assumed constant for the year between time *t* and *t* + 1, all continuous-time equations were numerically solved in finer bi-weekly time steps, thus achieving a satisfactory approximation of continuous time. For fitness optimization, we maximized expected lifetime energy available for reproductive investment. The predicted life histories are evolutionary optima, i.e. resulting from adaptive phenotypic plasticity that the species or population may harbour already or which might evolve as new adaptations given sufficient time for evolution to reach the new equilibrium and given that the ecology described by the parameter set remains constant. For results shown, we have simulated 1000 individuals following the optimal strategy but varying in the experienced stochastic environment. Population characteristics and their variability emerge, including growth trajectories, maturation age and size, reproductive investment and age and size structure, shown in the Results section. The critical assumption here is that individuals behave optimally as they interact with a particular environment, as well as in the new environments that are assumed to follow from climate warming.

The optimization algorithm finds the energy allocation (α) and foraging behaviour (*φ*) for each combination of state variables (age *A*, length *L* and food environment *E*) that maximize future reproductive output *R*(*A*, *L*), as follows:

(19) $$R(A,L)=\underset{\alpha ,\varphi }{\max}\sum P(E)[S(E)(R(A+1,\;L^{\prime }(E))+G^{\prime }(E))]$$

Here *R*(*A*, *L*) is the expected future reproductive output of an individual of age *A* and length *L* and is found through this recursive equation; *G*′ is reproduction between age *A* and *A* + 1 and thus influenced by the current food level, while *R*(*A* + 1,*L*′) is the future expected reproduction for the rest of the life given the new state *L*′. Everything is discounted by survival probability (*S*). As *L*′, *G*′ and *S* depend on the environment (*E*), the consequences are summed over the probability of experiencing food environment *E*, *P*(*E*), assuming that the fish use an optimal strategy in each environment *E*. The strategy parameters α and *φ* affect the new states *L*′ and *G*′ as well as the survival probability (*S*).

### Parameterization

Parameter values have been chosen to represent NEA cod in the Barents Sea (see Table 2 for values and references). In accordance with bottom temperatures quantified for Atlantic cod stocks by Drinkwater (2005), we use 4°C as the current mean temperature experienced by NEA cod, with annual temperatures following a sinusoidal function ranging from 3.5°C in March to 4.5°C in July (Supplementary Fig. S2). For different temperature scenarios, we vary the mean and keep the range of temperature seasonality constant. The model considers only adult females. Several parameter values are difficult or impossible to measure and were tuned in the spirit of pattern-oriented modelling (Grimm *et al.*, 1996, 2005; see Supplementary Figs S3–S7 for sensitivity analysis). We used size-at-age, age- and size-at-maturity, maximal length and weight, as well as total natural mortality to tune parameter values.

## Results

Our model predicts that climate warming has effects on growth, the foraging strategy and reproductive investment beyond what is expected from physiological considerations alone, in turn having consequences for natural mortality, stock reproduction and fitness.

**Figure 2:**
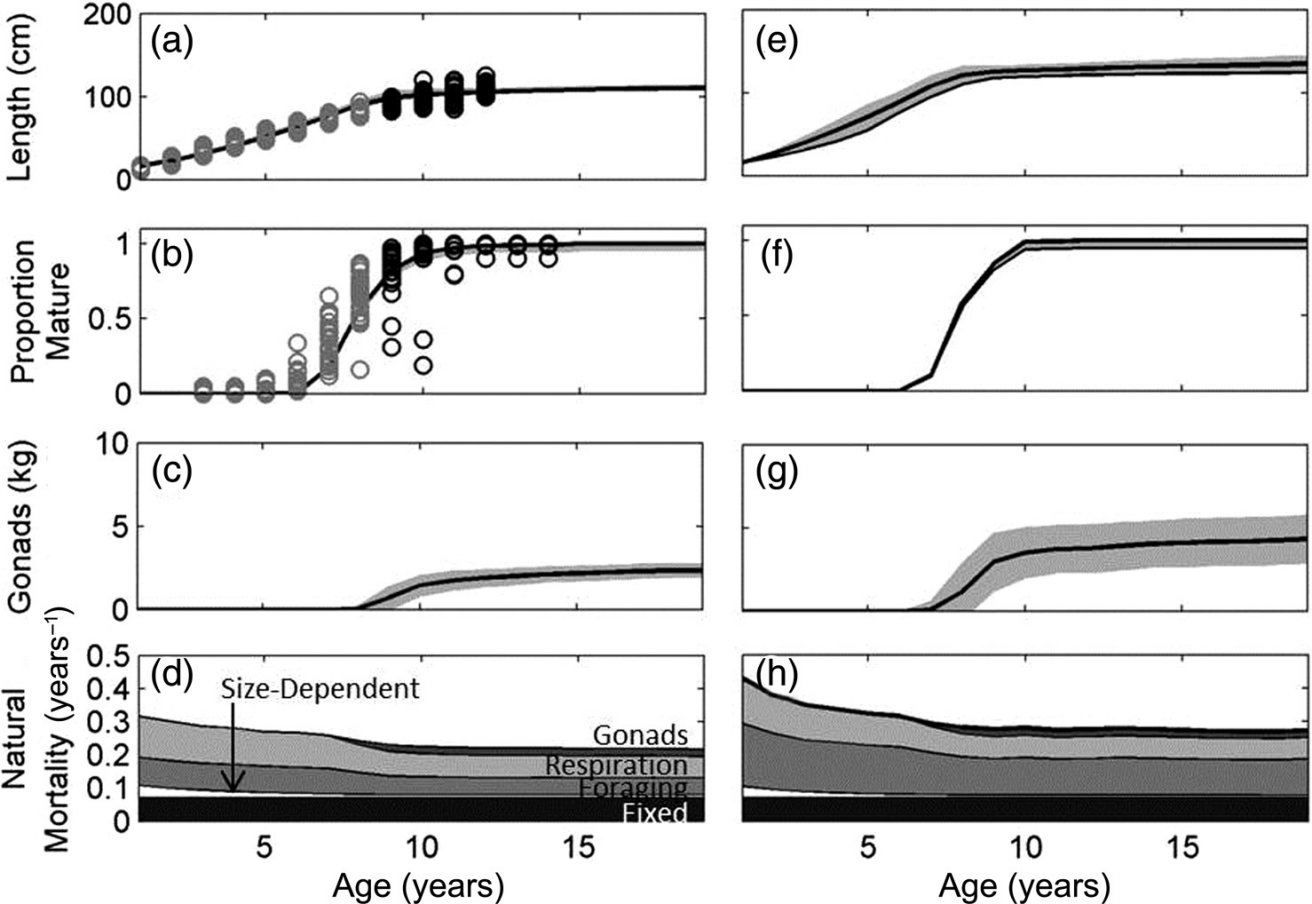
Predicted length-at-age (**a**) and proportion mature-at-age (**b**) compared with the International Council for the Exploration of the Sea (ICES) survey data from the Barents Sea (grey open circles) and Lofoten (black open circles; ICES, 2012). Predicted size of gonads (**c**) and annual rates of natural mortality (**d**) at current habitat temperature of 4°C as function of age. Panels (**e**)–(**h**) are equivalent to panels (a)–(d) but under a 2°C warming scenario. Thick black lines denote mean values and grey shaded areas denote within-population standard deviation due to environmental stochasticity.

We first validated model predictions against observed life-history characteristics of NEA cod at the current mean temperature (4°C; Fig. 2a–d). The predicted length-at-age and proportion mature-at-age both fell within the range of observed field data from the Barents Sea and Lofoten for the period 1980–2012 (Fig. 2a and b; ICES, 2012). Size of gonads-at-age was in the range of values reported in the literature (e.g. Nash *et al.*, 2008; Yaragina, 2010). The degree of fit between the model prediction and data for NEA cod (ICES, 2012) and the current life history for NEA cod was sufficient to proceed with these standard parameters when providing further climate comparisons.

Given that the model is based on mechanisms and processes, it also makes predictions for traits and characteristics that are harder to observe. In particular, it predicted the annual rate of natural mortality to be 0.23 year^−1^ at age 10 years (Fig. 2d), which is comparable to but higher than the value of 0.2 year^−1^ for ages 4 year and up used by the International Council for the Exploration of the Sea (ICES) in the stock assessments (ICES, 2012). A similar value of 0.25 year^−1^ was required when Jørgensen and Fiksen (2006) fitted a similar type of model to the same stock. Recent studies on Canadian cod stocks have suggested a higher and increasing rate of natural mortality, exceeding 0.6 year^−1^ in the most recent period (Swain, 2011).

To illustrate predicted changes, we show responses under a 2°C warming (Fig. 2e–h). In terms of length, the model predicted faster growth and a larger asymptotic size (size at age 10 years was 126 cm at 6°C compared with 106 cm at 4°C; Fig. 2e). Maturation age was unaffected by the 2°C warming (Fig. 2f), but gonads were predicted to more than double in weight, from a mean of 1.4 to 3.5 kg at age 10 years (Fig. 2g), not only because the fish were larger but also because the gonadosomatic index increased. This aligns with the general prediction of better recruitment in warm years for this stock (Planque and Frédou, 1999; Drinkwater, 2005). In addition, new strategies became optimal, which can be seen in how foraging mortality at age 10 years increased from 0.07 to 0.11 year^−1^, illustrating a shift towards more intense and risky foraging, which would yield more resources for growth and reproduction. This had consequences for total natural mortality, which at age 10 years was predicted to increase from 0.23 year^−1^ at 4°C to 0.28 year^−1^ at 6°C (Fig. 2h).

**Figure 3:**
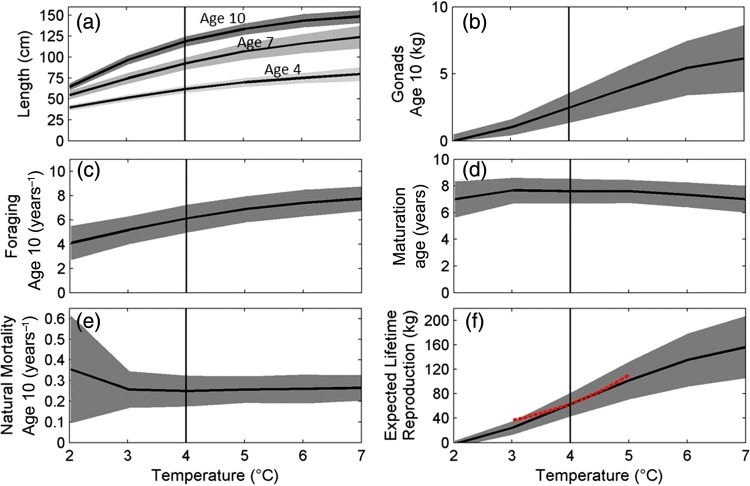
Predicted responses to annual ocean temperatures from 2 to 7°C. Phenotypic traits: body length (**a**) and gonad weight (**b**). Strategies: foraging (**c**) and age at maturation (**d**). Population-level consequences: natural mortality (**e**) and expected lifetime gonad production (**f**). In (f), the model's prediction of expected lifetime gonad production is compared with the population-level observation of recruitment (red dashed line) reported by Drinkwater (2005). The current mean temperature for North-East Arctic cod, 4°C, is indicated by black vertical lines. Thick lines denote the population mean value, while shaded grey areas show the within-population standard deviation due to environmental stochasticity.

A more detailed picture of how temperature shapes optimal life histories of NEA cod can be seen by examining model predictions for a wider range of temperatures, from 2 to 7°C (Fig. 3). In general, temperature responses were gradual; fish were predicted to grow faster and be more fecund at higher temperatures. The positive effect of temperature on growth rate is exemplified by increasing length-at-ages 4, 7 and 10 years (Fig. 3a) and increasing gonadal weight (Fig. 3b). To attain the faster growth, the cod exhibited increased foraging behaviour (Fig. 3c). Age of maturation remained unchanged over most of the temperature ranges, but was predicted to be slightly lower in both colder and warmer scenarios (Fig. 3d). All these changes sum up to consequences for survival and fitness. Over most temperatures, changes towards larger sizes, which would lower predation rates, were counterbalanced by more risky foraging strategies; total natural mortality consequently remained constant (Fig. 3e). Only at very cold temperatures did the model predict a higher natural mortality rate (Fig. 3e). Lifetime gonad production, a proxy for fitness, strongly increased with warmer temperatures over the entire range, with an expectation of ∼53% higher reproductive output at 6 compared with 4°C (Fig. 3f). We compare this individual-level prediction with the population-level observation of recruitment increasing by ∼40–50% per 1°C increase of sea surface temperature (Drinkwater, 2005; Fig. 3f, red dashed line).

## Discussion

In this paper, we focus on temperature-induced adaptations for NEA cod. We showed that aerobic scope provides important constraints for the plasticity and evolution of behaviour and life histories, and that natural selection may cause adaptations in a suite of traits that together determine fitness in new environments. Our model predicted that temperature will not only accelerate physiological processes, but will also select for faster life-history strategies within this stock, implying that the effects of temperature depend on ecological parameters and that phenotypic plasticity and genetic adaptation together may influence the rate and extent of change.

### Model assumptions

In addition to energetics, the key physiological mechanism in our model is constraints on aerobic respiration. A central role for aerobic scope was suggested by Fry (1971), and Priede (1985) interpreted consequences for adaptation and organism design. These perspectives have been elaborated by Pörtner (2010) when developing the OCLTT theory, which states that the capacity to provide oxygen (aerobic scope) has to support processes such as foraging, locomotion, growth and reproduction. Fry (1971) classified environmental factors based upon their influence on metabolic rate and aerobic scope, suggesting that both temperature and other stimuli can increase or decrease the capacity of the organism to provide oxygen (aerobic scope) and oxygen demand (metabolic rate; Fry, 1971; Pörtner and Peck, 2010). The organism's oxygen budget therefore has to be considered not only in terms of the role of temperature on physiological rates, but also through integration with ecological processes that have consequences for survival and reproduction and therefore fitness, potentially causing Darwinian evolution. Our model thus highlights how aerobic scope is finite, and therefore, that some degree of oxygen budgeting (Killen *et al.*, 2007) must take place to encompass key fitness-related processes. Thus, changes in temperature alter the capacity to provide oxygen, and one may expect adaptations of behaviour and life histories.

The OCLTT theory goes beyond earlier work by further summarizing state-of-the-art knowledge of how physiological stress responses translate directly into changes in aerobic performance (Pörtner, 2010; Pörtner and Peck, 2010). The OCLTT theory then makes what might be described as a leap of faith when it subsequently assumes that effects on fitness will follow the same temperature relationship as aerobic scope (Pörtner, 2010), without basing this on a mechanistic understanding. We model this missing link, by using aerobic scope as the key physiological constraint that defines key trade-offs that shape climate-driven evolution of behaviour and life histories.

The optimization method finds expected evolutionary optima but is ignorant about the rates of change and whether the optimal trait values can be reached. How fast traits will change depends upon the nature of the trait, i.e. whether it is behavioural or physiological, plastic or genetic. Behavioural traits such as those responding to a direct social cue (Huse *et al.*, 2002) or environmental stimuli may respond very quickly (Lima and Dill, 1990). Some phenotypically plastic traits may develop through ontogeny and depend on environmental experience and are likely to be slower, but may respond within a generation or lifetime of an organism (Hoffmann and Sgrò, 2011; Kopp and Matuszewski, 2014). Genetic traits are slower, requiring genetic evolution over multiple generations (Kopp and Matuszewski, 2014). The model does not distinguish between phenotypic plasticity and genetic change, but finds the optimal life-history strategies for a given set of parameters. One question is whether evolutionary changes can be fast enough to keep up with the decadal time scales of predicted temperature increases due to global change (IPCC, 2013). Evolution of life-history traits has probably occurred on decadal time scales in response to fishing (Jørgensen *et al.*, 2007; Darimont *et al.*, 2009; Devine *et al.*, 2012), and experiments also corroborate that life-history changes can be fast (e.g. Reznick, 1990; Conover and Munch, 2002). These data suggest that decadal evolutionary responses to ocean warming can occur if the selection pressure is sufficiently strong. In addition, adaptive phenotypic plasticity to temperature variation can induce responses that do not require genetic change and therefore can be faster. Quantitative genetic analysis within an animal model approach (e.g. Kruuk, 2004; Teplitsky *et al.*, 2008) may distinguish between plastic and genetic effects and has suggested that climate-related responses in mean body size of a bird population were probably due to phenotypic plasticity rather than micro-evolutionary responses (Teplitsky *et al.*, 2008).

### Model predictions

Within the temperature range of this study, the aerobic scope increases monotonously because the temperature of peak aerobic scope is 13–15°C for cod (Claireaux *et al.*, 2000). Our model thus predicts both increased growth rate and increased fecundity as a result of increased aerobic scope, which is consistent with studies by Michalsen *et al.* (1998) in which they found that mean length-at-age increased with increasing temperature for NEA cod aged 2–6 years. Likewise, Brander (1995) found temperature-dependent increases in weight-at-age for several cod stocks across the North Atlantic.

Our predictions and these observed increases in size-at-age at higher temperatures are contrary to the climate warming-induced trend towards smaller body size reported in several studies (Daufresne *et al.*, 2009; Cheung *et al.*, 2012). The reason for this might be that the NEA cod is at the lower end of the species' temperature range, where warming can be beneficial (Brander, 1995; Drinkwater, 2005; Hollowed and Sundby, 2014), while other studies commonly focus on species at the warmer end of their distribution because these are imperilled by climate warming. However, the predicted increases in growth and fecundity are based on the assumption that food availability in the Barents Sea remains constant with increasing temperature. A decline in food availability in the Barents Sea would negatively impact this stock, implicating energy allocation to growth and reproduction. Additionally, density-dependent effects may impede the benefits of increased growth rates by increasing competition for food. However, recent positive changes in food availability are evident in the Barents Sea, due to both larger ice-free areas and increased advection of zooplankton food from Atlantic waters into the Barents Sea (Dalpadado *et al.*, 2012; Kjesbu *et al.*, 2014). The NEA cod has increased in recent years, with unprecedentedly high spawning stock biomass, probably as a consequence of both increased temperatures, resulting in an extension of the stock's feeding area, and the implementation of a precautionary harvest control rule (Kjesbu *et al.*, 2014).

A bioenergetics budget has to balance energy use and energy acquisition, and this is often achieved through behaviourally mediated trade-offs with consequences for survival and fitness. Increased energetic requirements from resultant temperature increases may require an individual to forage more often or take higher risks to achieve its required rate of food intake. The metabolic rate of an individual could therefore be linked to the willingness to take risks whilst foraging (McNamara and Houston, 1986; Finstad *et al.*, 2007; Careau *et al.*, 2008; Huntingford *et al.*, 2010; Killen *et al.*, 2011). The utilization of resources requires prior physiological and morphological investments in searching for and handling prey, which are costly (McNab, 1988). In response to climate warming, both costs and efficiency of food acquisition may vary, potentially altering metabolic investment strategies (Crozier *et al.*, 2008).

In our model, an emergent pattern of higher risk acceptance and increased foraging behaviour was predicted with increasing temperature. Foraging behaviour is intrinsically linked to growth rate, being adaptive given the local environment while subjected to physiological constraints. This has also been demonstrated by counter-gradient variation in growth exhibited across latitude by Atlantic silversides (*Menidia menidia*; Chiba *et al.*, 2007). Similar results have also been found in European sea bass (*Dicentrarchus labrax*), where individuals with higher metabolic rates took greater foraging risks to meet their energy requirements (Killen *et al.*, 2011). Furthermore, in whole-lake manipulation experiments of rainbow trout (*Oncorhynchus mykiss*), increased rates of metabolism at higher temperatures resulted in increased feeding activity, interpreted as a compensatory mechanism through which individuals may maintain growth rate (Biro *et al.*, 2007).

Temperature can have important consequences for the energy allocated to somatic growth and reproduction. Our model predicted increased reproductive investment for NEA cod in terms of both gonadal weight and lifetime gonad production, a proxy for fitness, with increasing ocean temperatures. This is consistent with Yoneda and Wright (2005), who found that higher temperatures resulted in higher gonadosomatic index in first-time spawning Atlantic cod in the North Sea.

In a similar manner to the NEA cod, early-maturing cod stocks in the North-West Atlantic are suggested to benefit from increasing temperatures (Planque and Frédou, 1999; Drinkwater, 2005). In contrast, however, the faster-growing cod stocks in warmer regions are imperilled, and local extirpation of cod stocks in the Celtic Sea and English Channel is expected if current temperatures (11°C) increase above 12°C, which is predicted for these areas (Drinkwater, 2005). Warm-water stocks in the Irish and North Sea as well as Georges Bank are expected to decline owing to a reduction in recruitment with warming temperatures (Planque and Frédou, 1999; Drinkwater, 2005). For other cod stocks with more northerly distributions, individual growth rates may become faster, which is likely to increase productivity in these areas (Drinkwater, 2005). The picture is further complicated by the fact that individuals of the same stock may differ hugely in their experienced temperatures (e.g. Righton *et al.*, 2010), which might be due to variation in habitat choice or behaviour or perhaps that temperature preferences vary with physiological or reproductive state.

### Model omissions

While our model focuses only on effects of temperature, global climate change will probably be more diverse and have biological effects through other drivers, such as ocean acidification, hypoxia or ocean stratification, which we do not include in the model.

In responses to climate warming, species may adapt to the new environment or exhibit distributional range shifts (Burrows *et al.*, 2011). Isothermal habitats are shifting poleward in the marine environment (Easterling, 2000; Hughes, 2000; McCarty, 2001; Walther *et al.*, 2001, 2002; Drinkwater, 2005; Burrows *et al.*, 2011; Cheung *et al.*, 2012) as well as down the water column, whereby some fish species have moved to deeper habitats (Burrows *et al.*, 2011). We focus on how NEA cod may adapt and stay within their current distribution, but our model does not consider that they might move to new areas for feeding or spawning. Increased temperatures have been suggested to result in an extension of suitable feeding area for NEA cod (Kjesbu *et al.*, 2014), and currently, this seems to accelerate growth rates beyond what was predicted in our model.

For some species, changing geographical distribution may not be an option, and the dynamics one could expect in such a situation would be more similar to what we have modelled. For example, Baltic Sea cod are unable to inhabit waters further north due to unfavourable salinity conditions that have a negative influence on the viability of the eggs and larvae (MacKenzie *et al.*, 1996; Koster *et al.*, 2003). Likewise, the Mediterranean Sea, the Black Sea and the Gulf of Mexico are examples where a northwards distribution would be prohibited by land masses.

In addition, the model does not encompass changes in migration, for example, in the distance or timing of migration (Jørgensen *et al.*, 2008). The NEA cod are known to perform extensive, energetically costly migrations to and from their spawning grounds in Lofoten (∼800 km). The trade-offs between the costs and benefits of migration might crucially depend on body size (Roff, 1988) and condition (Block *et al.*, 2005), with the mass-specific cost of transport being lower with larger body size (Ware, 1978). For the NEA cod, our model predicted that larger body size was optimal at increased temperatures, and given that body size has consequences for swimming costs, one can expect that migration strategies might also evolve in response to climate, although this is not quantified in the present study.

Furthermore, we focus on the adult life-history response to climate warming. Technically, fish enter the model at age 1 year and there are no climate influences on the recruitment relationship between total egg production and juveniles. Of course, temperature may also have effects on early life stages (Planque and Frédou, 1999; Drinkwater, 2005; Ottersen *et al.*, 2006, 2013). Ottersen *et al.* (2013) found a positive temperature relationship in cold-water stocks of the North Atlantic and a negative relationship with warmer stocks, for both Atlantic cod and herring (*Clupea harengus*). Despite not studying early life stages explicitly, we provide predictions of total lifetime reproduction in terms of gonads, and compare this individual-level prediction with the population-level observation of increasing recruitment with increasing temperature (Drinkwater, 2005; Fig. 3f). At the same time, biophysical individual-based models for early life stages have suggested that higher temperatures are beneficial for developing NEA cod larvae (Vikebø *et al.*, 2005; Opdal *et al.*, 2011). Our predictions are consistent with the ∼40–50% increase in recruitment per 1°C increase in sea surface temperature (Drinkwater, 2005). Any positive effects of temperature on early life stages would add to the slope of recruitment vs. gonad production predicted by our model. The high degree of fit between Drinkwater's (2005) curve and gonad production in our model thus suggests that our model overestimates the temperature effect on gonad production or positive effects of temperature on early life stages have been overestimated or there are unknown negative effects of higher temperature that neither of these approaches has yet taken into account.

In the wider ecological perspective, climate warming may affect any part of the ecosystem, with consequences for predation rates, productivity, food availability and food-web dynamics (Fulton, 2010). We assume that all ecological components in the model remain constant when transitioning from the current environment to the new warmer environment. End-to-end models, although in early stages of development, can be used to assess the effects of climate change in marine ecosystems, and their strength lies in combining physical oceanography, biogeochemistry and organisms ranging from microbes to higher trophic levels (Kishi *et al.*, 2007; Rose *et al.*, 2010; Griffith and Fulton, 2014). Eventually, there is a need to couple models of changing physical and biotic environments to models of changing traits within species.

### Implications for conservation

Many fish populations, including NEA cod, have been exploited historically and are suggested to be particularly sensitive to the effects of climate change (Planque *et al.*, 2010). We predict a positive outlook for NEA cod in response to climate warming, which has important conservation implications for this stock. The situation can be different for stocks nearer the optimal temperature of aerobic scope or even beyond it. Modelling studies such as this one could be useful for management, for instance, in the Barents Sea Management Plan (Olsen *et al.*, 2007), for which the effects of climate are not included. Although the results predicted here are positive, they should not be taken out of context. Increases in growth and fecundity are conditional to food levels and other ecological factors, such as predation rates, remaining constant. If food levels were to decrease, it may have drastic consequences for this stock, with increased mortality following from more risky foraging strategies.

There is an expectation that northward movements of boreal, commercially attractive species will increase in the Barents Sea in coming years (Drinkwater, 2005; Stenevik and Sundby, 2007). As a consequence, the Barents Sea will be likely to receive more attention from the fishing industry (Christiansen *et al.*, 2014). Changes in fishing mortality may hinder any positive influence of climate on this stock. For example, the success of NEA cod recently was a result of a combination of increased feeding areas due to climate and the effective implementation of a precautionary harvest control rule (Kjesbu *et al.*, 2014). Therefore, if management plans are to be successful, multiple stressors should be taken into consideration within an ecosystem-based management approach.

### Conclusion

For the NEA cod, our model predicted the effects of climate warming to be positive; increased temperatures were predicted to select for faster life-history strategies, increasing growth rates, larger size-at-age and higher reproductive output without added mortality. In sum, higher temperatures are thus predicted to lead to higher expected lifetime gonad production and better recruitment. Our model fills in the gap between respiratory physiology as described by OCLTT theory and how it may influence fitness, revealing the underlying mechanisms and drivers of change in NEA cod.

## Supplementary material

Supplementary material is available at *Conservation Physiology* online.

## Funding

R.E.H. acknowledges funding from Nordforsk through the Nordic Centre of Excellence for Research on Marine Ecosystems and Resources under Climate Change (NorMER).

## Supplementary Material

Supplementary Data
