# Supplementary material for: Climate warming causes life-history evolution in a model for Atlantic cod (Gadus morhua)
Source: Conserv Physiol. 2014 Nov 4;2(1):cou050. doi: 10.1093/conphys/cou050 (PMC4806736; doi:10.1093/conphys/cou050)
Supplement: Supplementary Data [file supp_2_1_cou050__index.html]

Climate warming causes life-history evolution in a model for Atlantic cod (Gadus morhua) — Supplementary Data 

# Climate warming causes life-history evolution in a model for Atlantic cod (*Gadus morhua*)

## Supplementary Data

Supplementary Data

**Files in this Data Supplement:**

- Supplementary Data - Docx file
