# Supplementary material for: Climate warming causes life-history evolution in a model for Atlantic cod (Gadus morhua)
Source: Conserv Physiol. 2014 Nov 4;2(1):cou050. doi: 10.1093/conphys/cou050 (PMC4806736; doi:10.1093/conphys/cou050)
Supplement: Supplementary Data [file supp_cou050_cou050supp.docx]

**Supporting Information**

**Climate warming causes life history evolution in a model for Atlantic cod (*Gadus morhua*)**

*Rebecca. E Holt and Christian Jørgensen*

**Respiration Physiology**


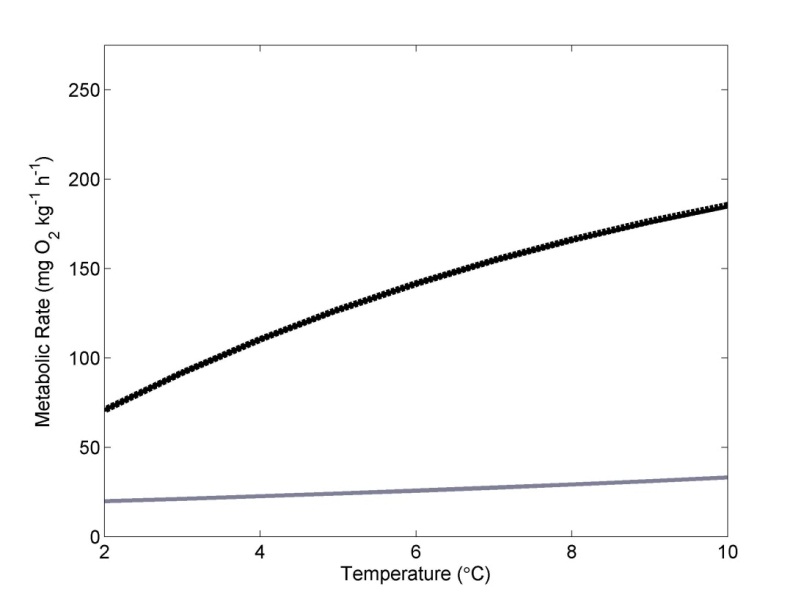


Figure S1. $B_{\mathrm{SMR}}$ (Standard metabolic rate) for a general teleost fish (Grey solid line) (Clarke and Johnston, 1999) and *V*max (Maximal aerobic metabolism) as per Claireaux *et al*., (2000) indicated by the black dashed line.

**Seasonality**

(S2) $\tilde{T}(y)=T+0.5\cdot T_{a} \cdot\cos(y-T_{p})\cdot2 \pi$

**
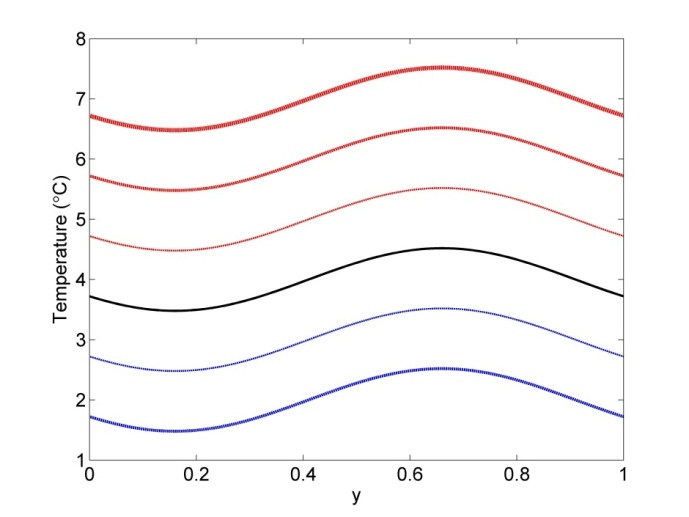
**

Figure S2. Temperature Seasonality. Black line indicates current temperature seasonality in the Barents Sea (ICES, 2012, Brander, 1995). Red dashed lines indicate increasing temperature scenarios in 1°C increments as simulated in the model. Blue dashed lines indicate decreasing temperature scenarios in 1°C increments as simulated in the model.

**Sensitivity Analysis**

1. **Respiration Mortality**


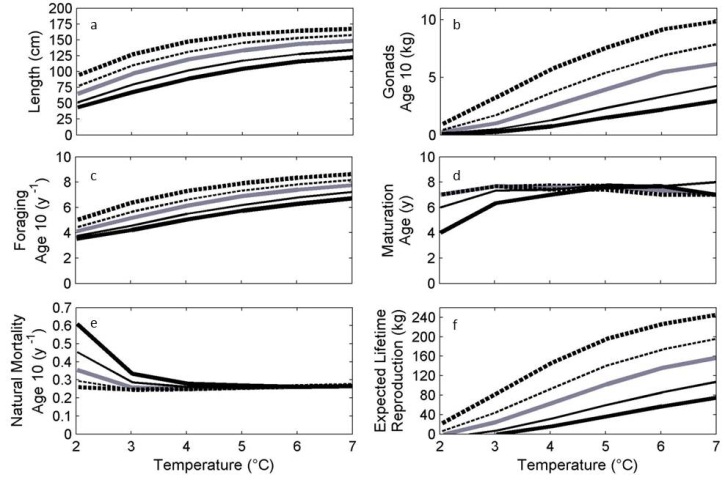


**Fig S3.** **Sensitivity analysis for varying the respiration mortality coefficient,**$\boldsymbol{c}_{\boldsymbol{respiration}}$**.** Predicted mean phenotypic traits: body length-at-age (a) and gonad weight-at-age (b). Strategies: foraging (c) and age at maturation (d). Population level consequences: natural mortality (e) and expected lifetime reproduction (f) given sensitivity to varying parameter values of respiration mortality coefficient. Central Grey line represents the parameter set used in the model to represent North-East Arctic cod. Different line styles denote varying parameter values (in percent of standard value): thick solid (200), thin solid (150), thick dashed (50), and thin dashed (75).

**
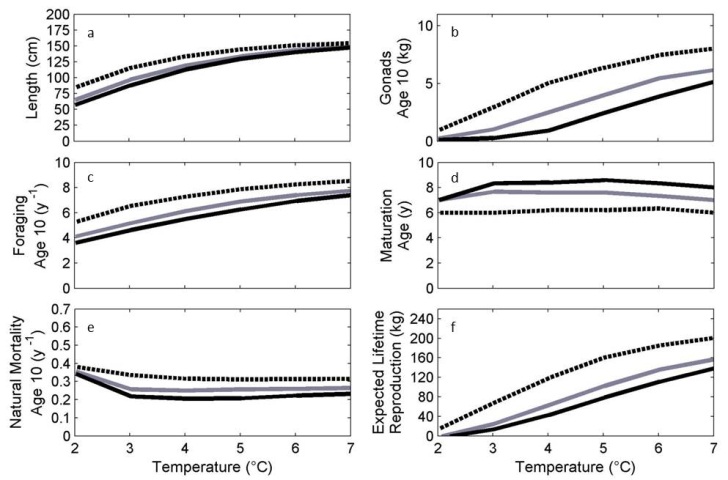
**

**Fig S4. Sensitivity analysis for varying the respiration mortality exponent,** $\boldsymbol{u}$**.** Predicted mean phenotypic traits: body length-at-age (a) and gonad weight-at-age (b). Strategies: foraging (c) and age at maturation (d). Population level consequences: natural mortality (e) and expected lifetime reproduction (f) given sensitivity to varying parameter values of respiration mortality exponent. Central Grey line represents the parameter set used in the model to represent North-East Arctic cod. Different line styles denote varying parameter values: thick dashed (exponent = 2), thick solid line (exponent = 4).

1. **Migration Distance**

**
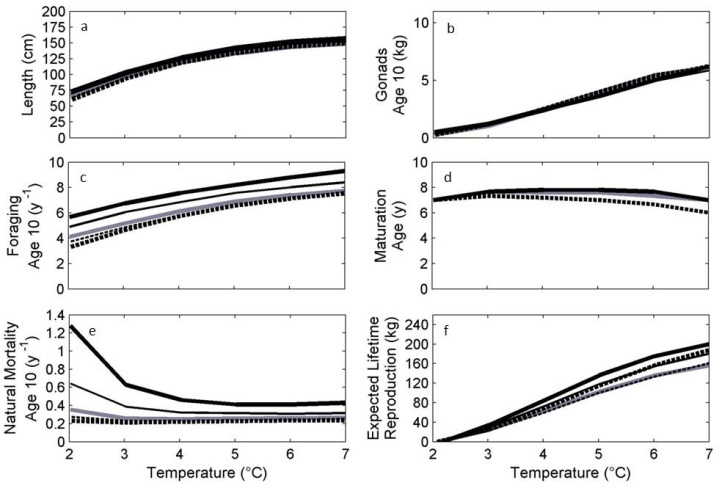
**

**Fig S5. Sensitivity analysis for varying spawning migration distance, *D_M_*.** Predicted mean phenotypic traits: body length-at-age (a) and gonad weight-at-age (b). Strategies: foraging (c) and age at maturation (d). Population level consequences: natural mortality (e) and expected lifetime reproduction (f) given sensitivity to varying parameter values of spawning migration distance. Central Grey line represents the parameter set used in the model to represent North-East Arctic cod. Different line styles denote varying parameter values (in percent of standard value): thick solid (200), thin solid (150), thick dashed (50), and thin dashed (75).

1. **Fishing Mortality**

**
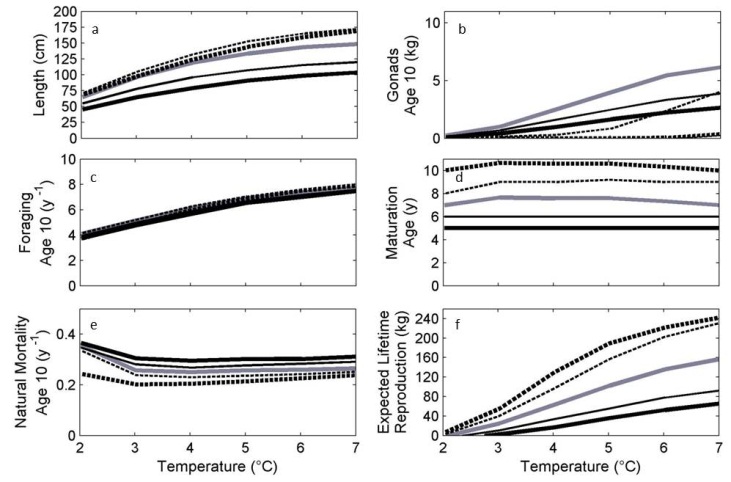
**

**Fig S6. Sensitivity analysis for varying fishing mortality, *F*.** Predicted mean phenotypic traits: body length-at-age (a) and gonad weight-at-age (b). Strategies: foraging (c) and age at maturation (d). Population level consequences: natural mortality (e) and expected lifetime reproduction (f) given sensitivity to varying parameter values of fishing mortality. Central Grey line represents the parameter set used in the model to represent North-East Arctic cod. Different line styles denote varying parameter values (in percent of standard value): thick solid (200), thin solid (150), thick dashed (50), and thin dashed (75).

1. **Temperature Amplitude**

**
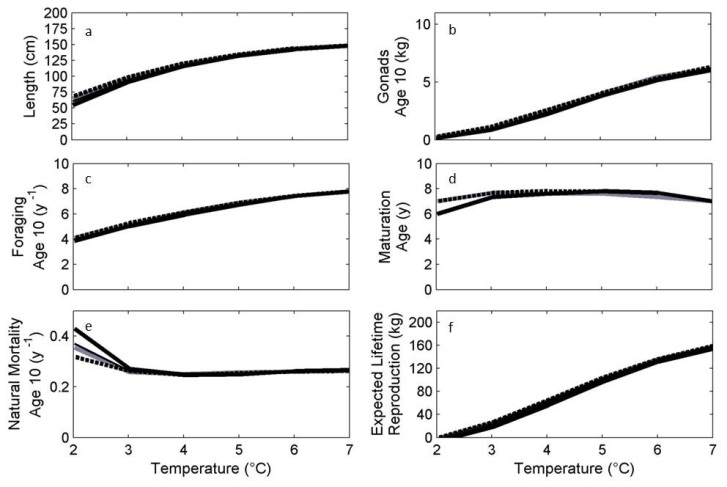
**

**Fig S7. Sensitivity analysis for varying Temperature amplitude,**$T_{a}$**.** Predicted mean phenotypic traits: body length-at-age (a) and gonad weight-at-age (b). Strategies: foraging (c) and age at maturation (d). Population level consequences: natural mortality (e) and expected lifetime reproduction (f) given sensitivity to varying parameter values of temperature amplitude. Central Grey line represents the parameter set used in the model to represent North-East Arctic cod. Different line styles denote varying parameter values (in percent of standard value): thick solid (200), thin solid (150), thick dashed (50), and thin dashed (75).
